# Temptation at the school fence: a qualitative exploration of the impact of external food outlets on the school community

**DOI:** 10.1186/s12889-026-26917-0

**Published:** 2026-03-13

**Authors:** Sharonna Mossenson, Frith Klug, Jacinta Francis, Siobhan Hickling, Lukar Thornton, Gina Trapp

**Affiliations:** 1 Nutrition and Health Innovation Research Institute, School of Medical and Health Sciences, Edith Cowan University, 270 Joondalup Drive, WA 6037 Joondalup, Australia; 2 The University of Western Australia, Perth, WA Australia; 3 The Kids Research Institute Australia, Perth, WA Australia; 4 Cardiovascular Epidemiology Research Centre, School of Population and Global Health, The University of Western Australia, Perth, WA Australia; 5 Department of Marketing, University of Antwerp, Antwerp, Belgium

**Keywords:** Food environment, schools, adolescents, food choice

## Abstract

Retail food outlets are commonly located in close proximity to schools, providing students with opportunities to purchase and consume food enroute to or from school. These outlets are typically unhealthy and disproportionately clustered near schools, and this trend has been increasing over time. While quantitative studies have established associations between school food environments and adolescent dietary behaviours, little is known about how school personnel perceive and experience their impacts. This qualitative study explored perspectives of school personnel (*n* = 9) from a secondary school in Perth, Western Australia, located 150 m from two retail food precincts (areas with a high concentration of food outlets, such as fast-food outlets, restaurants, cafés, and convenience stores). Semi-structured interviews were conducted, transcribed verbatim, and analysed thematically. School personnel described the nearby outlets as exerting a strong influence on students’ food choices, behaviours, and routines before and after school. Participants reported that nearby outlets increased students’ consumption of unhealthy food and drinks, contributed to classroom disruption, and undermined the school's health promotion efforts. The outlets also influenced the dietary practices of school personnel and increased their workload through the enforcement of school policies concerning external food. Broader community impacts, including loitering and shoplifting, created reputational issues for the school. While employment opportunities were acknowledged, overall sentiment from school personnel was overwhelmingly negative. Participants advocated for planning regulations to restrict the development of food outlets near schools and called for alternative youth-focused community infrastructure. Policy reform to limit outlets near schools, supported by cross-sector collaboration between education, health, and urban planning, is urgently needed to create healthier school food environments.

**Supplementary Information:**

The online version contains supplementary material available at 10.1186/s12889-026-26917-0.

## Introduction

Retail food outlets are commonly located in close proximity to schools, providing students with opportunities to purchase and consume food and beverages before, during, or after school [1]. Fast food outlets and convenience stores are often disproportionately clustered around schools across many global contexts [2, 3] and the density of these types of food outlets has been increasing over time in countries such as the United States, United Kingdom and in parts of Europe [4–6].

For example, a recent Australian longitudinal study found an almost 80% increase in the number of food outlets near secondary schools in regional and metropolitan New South Wales over a 17-year period, with most classified as unhealthy (e.g., restaurants, cafés, fast-food outlets and takeaway stores [7]. Cross-sectional data from Western Australia (WA) found that nearly 90% of Perth schools had a fast-food outlet within one kilometre, and 44% of schools, within 400 m; with secondary schools more likely to be near multiple outlets [8]. Similar patterns are evident in Victoria and South Australia, where fast-food outlets are more concentrated around secondary schools, particularly those in disadvantaged areas [9, 10]. Collectively, these findings reflect an obesogenic food environment that encourages and normalises unhealthy dietary behaviours among students.

Routine exposure to obesogenic food environments is associated with a higher frequency of unhealthy food purchases among adolescents on their way to and from school [2]. In a WA study, nearly half of secondary school students reported purchasing unhealthy food from nearby outlets each week, with purchase frequency linked to the density of major fast-food outlet chains near schools [11]. These purchasing patterns are further reinforced by adolescent students’ increased autonomy and greater financial independence [12, 13].

In addition, frequent exposure to and consumption of unhealthy food in the school food environment likely contributes to the development and normalisation of poor dietary behaviours, helping to explain the associations observed between the proximity and density of fast-food outlets near schools and higher rates of overweight and obesity among students [14, 15].

Despite extensive quantitative research on the clustering and impact of fast-food outlets around schools, there remains a qualitative research gap that captures school personnel perspectives on how the external school food environment impacts the school community. School personnel, such as the school leadership team (e.g., Assistant Principal, Dean of Studies), classroom teachers, specialist subject teachers, school youth workers and canteen staff, can provide valuable insights into how external outlets shape students’ food choices, school food practices and broader health promotion efforts. Therefore, understanding school personnel perspectives is crucial for informing local policy and school-level interventions, as they can elucidate barriers and unintended consequences, as well as highlight opportunities for policy and practice change. The existing qualitative literature is limited, often outdated, restricted to specific groups of school personnel or focused primarily on the internal school environment [16–18]. This leaves an important evidence gap regarding how school personnel perceive and navigate the external food landscape and its implications for equity, student health behaviours and school-based health promotion efforts. Because they interact with students throughout the school day, school personnel can offer insights that extend beyond observational data, providing a deeper understanding of students’ food experiences and how food environments influence students and school life. The purpose of this study was to adopt a qualitative approach to explore the perspectives of school personnel on the impact of the external school food environment on the school community, providing context-rich insights to inform future policy and intervention design.

## Methods

### Sampling and recruitment

In-depth interviews were undertaken between June and July 2024 with school personnel (*n* = 9) from an independent public co-educational secondary school located in the outer metropolitan area of Perth, Western Australia. Based on its Index of Community Socio-Educational Advantage (ICSEA), the school sits close to the national average of 1000, indicating that the student population is broadly reflective of the wider Australian secondary school population in terms of socio-educational advantage. The school was purposely selected due to its close proximity (approximately 150 m) to two retail food precincts, one of which was newly constructed and opened in 2021. Together, the two food precincts comprised ten external food outlets (fast-food/take-away outlets (*n* = 6), convenience store (*n* = 1), supermarket (*n* = 1), restaurant (*n* = 1), café (*n* = 1). A canteen (i.e., cafeteria) was also located within the school grounds.

The school principal provided consent for the school’s participation in the study and appointed a study co-ordinator to support the recruitment of staff. All school personnel were invited to participate voluntarily and were provided with a detailed information sheet and consent form. Participants included members of the school leadership team (e.g., Assistant Principal, Dean of Studies), classroom teachers, specialist subject teachers, school youth workers and canteen staff. Informed written consent was obtained prior to participation and there were no incentives provided. The study was approved by Human Research Ethics Committees at the University of Western Australia (Reference no. 2020/ET000264), Edith Cowan University (Reference no. 2024-05646-TRAPP); and the WA Department of Education (Reference no. D23/1309869).

### Data collection

A semi-structured interview guide was developed by the research team specifically for this study, with expertise in nutrition, food environments and qualitative research. The interview guide contained demographic questions (i.e., gender, length of time at the school, position at the school) as well as open-ended questions about (i) the school food environment; (ii) the impact of external food outlets on the school community; and (iii) existing and potential strategies to improve the school food environment. The interview guide is available as an additional file [see Additional File 1].

Interviews were conducted either via videoconference or face-to face, facilitated by SM and FK, respectively. Eight of the nine interviews were audio-recorded, while one was recorded in written format as the participant did not give consent to being audio-recorded. Interviews ranged in duration from 22 to 73 min. Data collection and analysis occurred concurrently and concluded when no new codes emerged.

### Data analysis

The audio recordings were de-identified and transcribed verbatim by SM with all transcripts subsequently reviewed by SE to ensure accuracy. Data were analysed using Microsoft Excel (Microsoft Corporation 2024) and applied thematic analysis [19], employing a combined inductive-deductive approach grounded in both the data and relevant literature. Initial coding and theme development were undertaken by SM and FK, two researchers with over 10 years of qualitative research experience. These researchers and participants were not known to one another. To enhance credibility, SM and FK independently coded a subset of five transcripts. Discrepancies were discussed collaboratively with the broader research team, leading to refinement of the coding framework and consensus on final themes, categories and codes. Some codes were applied across multiple categories to capture their occurrence in different contexts. Illustrative quotes from school personnel were selected to exemplify each category. The names of food outlets have been replaced with generic descriptions (e.g., convenience store) in the quotes to avoid identifying the outlets and location. The Consolidated Criteria for Reporting Qualitative Research (COREQ) guided the reporting of this study [20].

## Results

Nine school personnel participated in individual in-depth interviews. The sample characteristics are presented in Table 1.

**Table 1:** Characteristics of school personnel (*n* = 9)

Characteristic	Number of participants *n* (%)
Gender	
Male	2 (22)
Female	7 (78)
Years employed at school	
< 1 year	2 (22)
1–2 years	1 (11)
3–4 years	4 (45)
> 5 years	2 (22)
Position at school	
Leadership role	2 (22)
Specialist subject teacher	4 (45)
Other (e.g. youth worker, canteen manager)	3 (33)

Table 2 presents the overarching themes, categories and codes that emerged from the in-depth interviews.

**Table 2:** The impact of the external food environment on the school and surrounding community: Themes, categories and codes

Theme	Category	Code
Impact of food outlets located near schools	Impact on students	Food access
		Eating habits
		Built environment
		Advertising and promotion
		Smell
		Employment opportunities
		Social ties
	Impact on school personnel	Food access
		Convenience
		Eating habits
		Advertising and promotion
	Impact on the school	Classroom behaviour
		Undermining school curriculum
		Canteen
	Impact on the community	Anti-social behaviour
Strategies to support students to make healthier food choices	Government strategies	Planning regulation
		Provision of community space
	School strategies	External food policy
		Home economics and nutrition education classes
		Afterschool academies and clubs

### Theme 1: Impact of having external food outlets located next to schools

Theme 1 explores school personnel’s perceptions of the nearby external food outlets and their perceived impact on the school community.

### Impact on students

School personnel described a range of ways in which the external food environment impacted students.

School personnel employed at the school prior to the establishment of the newly constructed external food precinct noted that its introduction had a clear impact on students’ food access:

*“Having been here before when it was just a ‘sandpit’ [vacant block of land]*,* to now*,* students are always walking across from [the supermarket] or [fast-food outlet]… and they’re always picking up huge bottles of soft drink and massive bags of chips … the other day I saw three girls walking with soft-serve [ice-cream] cones at 8:00am in the morning”* (School Personnel #3).

This contrasted with students’ behaviour prior to the opening of the new food outlets. At that time, school personnel recalled that students typically congregated on the school oval, which served as a social gathering space:

“*It was a meeting point for some of the kids…they would just go with their bikes and sit*” (School Personnel #3).

Since the development of the nearby retail precinct, however, school staff observed a noticeable shift in behaviour, with students now spending their time at the shopping area instead.

School personnel expressed concern about the ease of access students had to the external food outlets, suggesting that their proximity influenced students’ routines before and after school:

“*We are literally a stones-throw away from the shopping centre…we’ve got [a supermarket]*,* there’s a [convenience store] there…so you know [students] have easy access…within a couple of minutes’ walk of the school*” (School Personnel #6).

*“You often see [the students] arrive to school and meet their friends at the front of the school and then walk back to [convenience store] or walk across the road to [the supermarket]*,* get [junk food]*,* and then consume it on the walk back to school…and I also see it after school”* (School Personnel #4).

*“Because of the accessibility and it’s morning time*,* [the students] might grab something before they head into the school…”* (School Personnel #8).

The ease of access to external food outlets had a direct influence on students’ eating habits, with many school personnel observing that the students were partial to: “*the Big Three”*, which comprised the convenience store, one of the fast-food outlets and the supermarket.

The ubiquity of energy-dense, nutrient poor food available at the nearby food outlets influenced students’ eating habits:

“*Kids often come to school with some sort of large*,* sugary drink before the day’s even started…which they’d usually buy from [the supermarket] or it’s not unusual to see kids with slushies [frozen sugar sweetened beverage] from [the convenience store]*” (School Personnel #3).

*“Kids are having [fast-food outlet] almost every morning…”* (School Personnel #2).

*“I always see students walking down with the slushies from [convenience store] before school…or they’ve got [fast-food outlet’s] soft serve cones”* (School Personnel #3).

These observed behaviours were seen as detrimental to students’ health and learning, and school personnel felt this was due to the influence and close proximity of the outlets:

*“It’s not a positive that [the external outlets] are so accessible…. if they weren’t so close*,* then perhaps students wouldn’t be making choices to purchase those types of foods*” (School Personnel #8).

Compounding students’ access to external food outlets were aspects of the built environment, such as the co-location of bus stops and food outlets:

“*…the bus stops are right next to [the food outlets]*” (School Personnel #7), so after school, “*often kids will meet their friends…go across the road to [the food outlets] …and then eat as they walk back to the bus stop*” (School Personnel #4).

The timing and regularity of buses also facilitated this afterschool routine:

“*The buses come pretty regularly after school*,* the kids can always wait for the second bus*,* go and hang out at the [supermarket] or [fast-food outlet] or [convenience store] and then come straight back [to the bus stop]*” (School Personnel #3).

Walkways were an additional built environment element that further enabled students’ access to the outlets:

“…*the actual path and the zebra [pedestrian] crossings take them directly past everything [the outlets] …*” (School Personnel #2).

School personnel also described how the pervasiveness of advertising and promotion amplified the students’ exposure to and use of the outlets:

“*I’m just obviously aware that students get bombed with…social media. You go basically in proximity of something these days. And next thing you know*,* you’ll be getting fed ads*” (School personnel #6).

“*[Advertising] is so close and so in their face*,* it encourages the students to access those sorts of things [the food outlets]”* (School personnel #7).

Likewise, the smell of the nearby outlets was considered an additional cue influencing students. One participant suggested that the smell alone could act as a trigger, prompting students to seek out the outlets, even without feeling hungry:

“*You can smell the fast-food cooking. It’s a significant issue. Pavlov’s Dog reaction*” (School personnel #1).

Another participant highlighted how smell further added to the sensory appeal of the outlets:

“*You can definitely smell it when they’ve got the deep fryers on. Probably more after school…there’s so many things luring these kids in*” (School personnel #3).

In contrast to the negative sentiments expressed by school personnel about the location and impact of the outlets, they did comment on the employment opportunities that the outlets offered the students:

*“A lot of [the students] have part time jobs there …they love working there*,* so I’m happy they’ve got employment”* (School personnel #3).

The food outlets were also described as a key space for students to socialise, providing an off-campus setting where they could gather with friends outside of school hours, with school personnel suggesting that the social ties with peers also influenced students’ purchasing habits:

“*Peer relationships are definitely a massive factor in how students make decisions…and I definitely think that peer relationships impact on students’ decisions around food*” (School personnel #8).

“*[Going to the food outlets] is just like a social thing…it’s how they socialise with each other*” (School personnel #3).

“*A lot of [students] use [the external food outlets] as a meeting place*,* so whether they’re buying from there or not…a large proportion of students would head that way anyway*” (School personnel #7).

“*It’s a destination…it’s part of a pre-school social ritual*” (School personnel #4).

### Impact on school personnel

School personnel described a range of ways in which the external food environment influenced school staff.

Regarding food access, school personnel utilised the external outlets for grocery shopping and socialising:

“*[Food and retail precinct] are less than a 200-metre walk*,* which benefits me when I do my afternoon shopping*,* or my weekly shop*” (School personnel #2).

“*Staff tend to use the local facilities*,* particularly [fast-food outlet] a lot. It is social because one person might be like*,* “oh*,* I’m going to go get something from [fast-food outlet] …do you want something?“…they go on their break*” (School personnel #8).

In particular, the nearby supermarket was seen to offer a level of convenience for school staff, serving both personal and professional needs:

*“I love having shops so close to school because I can click [order groceries] … to be ready at 3:00 o’clock…and they [the supermarket] send me a text message as I’m walking out…it’s very convenient”* (School personnel #4).

“*We spend over a grand [$1000] a week on ingredients for the kids to cook with and it all gets sent from [the supermarket] and it’s definitely convenient…if we need an extra bag of flour or we need a couple of spring onions…*” (School personnel #3).

Despite the perceived convenience, school personnel expressed ambivalence, describing the outlets as easily replaceable:

“*It would be no skin off my [nose] you know*,* personally it would be no difference to me. I can always ‘Click and Collect’ somewhere else*” (School personnel #2).

*Personally*,* I do not use the outlets closest to the school*,* because when there’s 1000 students roaming in the area*,* it’s not exactly an easy time to get anything…there’s probably somewhere closer to my house and I get to avoid the children*” (School personnel #5).

The school personnel noted that the proximity of the outlets influenced their own eating habits as well as those of other staff members:

“*I can’t lie*,* sometimes I’m really disorganised in the morning*,* and I go past and get [breakfast item] as do heaps of the other teachers…* (School personnel #3).

“*For my own healthy eating habits it is dangerous having [fast-food outlet] across the road*,* because at the end of a really long day*,* you’re like*,* oh go on*,* I’ll just go get some chips…and I’ll usually drive through*” (School personnel #4).

“*Even with my co-workers*,* like the [fast-food outlet] app…we’ll talk about deals that we’ve got on there or if we’re talking about lunch*,* like I know that some of my co-workers will walk over and get [fast-food] for lunch*” (School personnel #7).

School personnel were acutely aware of the various forms of advertising and promotion used by the nearby food outlets, with the outdoor signage standing out as particularly prominent and influential:

“*The [fast-food outlet’s] 30 metre pole is right out the front out there*” (School personnel #2), which “*you can see from all the two stories [buildings] in the school*” (School personnel #7).

“*The [fast-food outlet] has got the LED sign that promotes all their specials…and the bus stops*,* have got advertising…all of their [fast-food outlet] signage is huge*,* you can’t miss it*” (School personnel #3).

### Impact on the school

Many school personnel expressed concern about the perceived impact of students consuming food purchased from nearby food outlets on their classroom behaviour:

“*We have quite a few kids that just come in*,* like bouncing off the walls and I can guarantee they’ve probably been at the shops before school*” (School personnel #3).

The consumption of energy drinks was considered particularly problematic:

“*It’s very counterproductive to see them [students] with those high sugar items and energy drinks that would affect their learning…I see a lot of you know*,* particularly the younger kids walking into a classroom and not particularly paying much attention*,* not being in a position to pay much attention*” (School personnel #2).

“*I teach a program… for highly disengaged kids… it’s not unusual for some of those kids to arrive to school and have just had like*,* a large [energy drink] for breakfast that they bought at [the supermarket] …and that has a really big impact on their behaviour and engagement in the classroom*” (School personnel #4).

“*I definitely think the energy drinks play a massive part in the students not being able to concentrate and the increase of [disruptive] behaviours…because they’re struggling with concentration*,* and it [energy drinks] increases the disruptions in the classroom*” (School personnel #7).

Several school personnel expressed concern that the accessibility and appeal of the external outlets undermined the school curriculum and the school’s efforts to promote healthy eating:

“*I can go on and on to these kids about how much sugar is in soft drinks and what it does to your teeth and show them clips from that sugar film*,* where they have the kids with the Mountain Dew mouth…and they’re like*,* “oh my god”…but does it stop their behaviours [when we have all those foods so close by]? I don’t think so*” (School personnel #3).

“*It’s very tempting*,* like no matter how much information we have*,* like that temptation and at this age*,* I guess students can be a bit more impulsive in their in their decision making. Even armed with the information*,* temptation and accessibility definitely doesn’t help*” (School personnel #7).

School personnel also highlighted that students were still developing their nutrition literacy, potentially making them more vulnerable to unhealthy choices at the external outlets:

*“…developing their understanding of nutrition…and what’s healthy*,* not healthy…and there’s not much selection of nutritious food”* (School Personnel #8).

The school canteen operating from within the school was directly affected by the presence of the nearby food outlets, with school personnel commenting on the noticeable shift in students’ purchasing behaviour:

“*…sales went down [when the fast-food outlet opened] …*,* it was new*,* it was enticing. The kids wanted to meet there after school*” (School personnel #9).

Although the school maintains a ‘closed gate policy’, where students are prohibited from leaving the school grounds during the school day, school personnel observed that students often spent money that was intended for the canteen at nearby outlets before arriving to school:

“*I see the students walk over there with their lunch money pre-spent in the mornings on chips*,* and chocolate and cool drinks*,* and in particular*,* energy drinks*” (School personnel #2).

“*[Fast-food outlet] have their specials like $5 for a burger and chips…and the students keep their money till after school and get one of them [specials]*” (School personnel #9).

In response, the canteen adopted a range of strategies to try and compete with the pricing and appeal of the nearby outlets:

“*The hashbrowns came on the menu when [fast-food outlet] opened…so I bought smaller ones so we could sell them cheaper…we also sell fruit juice slushies*,* but because they’re pure juice they’re quite expensive…2.50. And you get them for $1 at [the convenience store]*” (School personnel #9).

### Impact on the community

School personnel described how the large number of students leaving school at the end of the day could be overwhelming, and often disruptive for the broader community:

“*You have 1500 students that are leaving school at 2:40pm. There has been complaints from the shopping centre*,* the [supermarket]*,* as well as the [fast-food outlet] about the behaviour of students. There is shoplifting*,* and disrespectful behaviour. It does become sort of a loitering spot where students will stay in these areas for a very long period after school has closed just because it’s in their neighbourhood*” (School Personnel #5).

Other interviewees echoed these concerns and further highlighted additional incidents of anti-social behaviour with broader implications for the community. These included reports of loitering, shoplifting, vandalism and general misconduct in and around the retail precinct:

“*When school finishes and you have 1500 kids leave school…they come into close contact with community. It can play out all sorts of different ways. Kids behaving badly. Kids stealing. Somebody in the community threatens and assaults a child. It can just be 50 kids inside the [fast-food outlet] and not listening to staff*” (School Personnel #1).

*“…loitering… amongst other things*,* but lots of theft as well. …[the supermarket] in particular have found theft*,* but [the fast-food outlet] found a lot of damage*,* including in their bathrooms”* (School Personnel #2).

*“We have had at times*,* you know*,* notifications around school that students are banned from [the supermarket] …we had same similar issues with the [convenience store] too…just big groups. Well*,* shoplifting is actually part of it*,* but big groups of kids hang around there and*,* you know*,* using it as meeting place and that*,* and that can tend to put off other people from using those facilities”* (School Personnel #6).

“*Even like the community members*,* like you’ve got young kids…and you’ve got a group of rowdy teenagers*,* it’s not the most pleasant experience*” (School Personnel #7).

A range of strategies were implemented by the external food outlets in collaboration with the school in an effort to manage students’ anti-social behaviour:

“*There used to be a lot of anti-social behaviour that would go on there and they’d [food outlets] would have to shut it [the food outlets] to the students*” (School Personnel #3).

“*[The fast-food outlet]*,* I know they banned our kids from coming in. It was only like a certain number…because of the destruction that they were causing…and they’ve [the supermarket] got regulations on how many of our kids can go in at one time as I know in the past there has been massive issues with shoplifting*,* particularly like the lollies and confectionary*” (School Personnel #7).

One participant observed that these behaviour management strategies weren’t always effective, given the sheer number of students and required law enforcement to attend:

“*The students just go en masse and it’s just this gang mentality where they wouldn’t move on*,* they wouldn’t do what they were asked to do by a staff member at the establishment…there was even police called a couple of times too for those incidents*” (School Personnel #2).

Many school personnel described the undue stress and added workload that the behaviour management strategies placed on both outlet and school staff:

“*It’s quite stressful for a lot of the workers [of the outlets] because they count our kids in and they count our kids out and it adds a lot of unnecessary stress*,* especially to the [supermarket] workers*” (School Personnel #7).

“*The school gets calls to lend assistance to local business and we do that because we want kids to behave well in the community…we worked hard to put governance around our expectations of kids when [fast-food outlet] was first established*” (School Personnel #1).

School personnel expressed concern that student behaviour in and around the external outlets negatively affected the school’s reputation within the community:

” *I think that there’s some implications around how our school is sort of presented to the public when there are large groups of students in uniform hanging out at those locations*” (School Personnel #2).

“*It brings the school into disrepute*” (School Personnel #7).

### Theme 2: Strategies to support students to make healthier food choices

Participants described a range of existing and proposed strategies, led by both government and schools, designed to support students in making healthier food choices.

### Government strategies

Many school personnel emphasised the need for government action to restrict the establishment of external food outlets near schools through planning regulation:

“*I would like to think that they [government] could bring in some sort of legislation that certain fast-food outlets can’t be built within a certain radius of schools*,* and anywhere there’s large groups of young adults*,* kids meeting*,* youth centres*,* things like that*” (School Personnel #3).

“*I think it’s a planning thing that needs the state government and local governments to come into it…having some sort of regulation around what can and can’t be literally across the road from a school*” (School Personnel #4).

“*In an ideal world*,* there would be clear distance limitations between an outlet [and a school]. To make it*,* not visible at least. So maybe that could be planning influence they [the government] could have*” (School Personnel #6).

Several school personnel were incredulous that planning regulation did not already exist. Some raised ethical concerns about commercial interests being prioritised over community wellbeing:

“*I can’t even believe that they’re allowed to open a [fast-food outlet] directly across from the school*,* I can’t believe there’s no legislation around it. Just seems so ridiculous… it’s all business*,* smart business*,* but ethical [business]? It [outlets close to schools] feels really wrong* " (School Personnel #3).

“*The cynic in me could say that big business is in it for the short-term gain*,* and they [government planners] don’t want to rock the boat and would prefer to sacrifice the long-term health [of children]*” (School Personnel #1).

“*If it was a school in a higher socioeconomic area*,* I don’t think parents would stand for*,* well*,* the proximity of [the convenience store] and [the fast-food outlet]*” (School Personnel #4).

School personnel advocated for the development and provision of community spaces near schools, such as youth centres or libraries, instead of retail precincts. These alternatives were perceived as more constructive, health-promoting environments that better support students’ wellbeing and social development:

“*They [should] put some sort of a youth centre or something over there* " (School Personnel #3).

“*I’d prefer it [retail precinct] be like a library* " (School Personnel #4).

### School strategies

While the proposed government-led strategies focused on *preventing* issues by avoiding the development of food outlets near schools, school personnel acknowledged that such changes are not feasible where retail precincts already exist near schools. In these contexts, school staff identified a range of strategies being implemented at the school level to help minimise the impact and potential harms associated with nearby food outlets.

In an effort to limit students bringing unhealthy items, particularly energy drinks, onto the school grounds, the school introduced an external food policy to discourage the consumption of food from nearby outlets:

“*The school has a policy that food from the outlets is not allowed*,* and we will confiscate*” (School Personnel #1).

However, many school personnel described the challenges in enforcing and monitoring the policy, particularly given the number of students at the school and when faced with scenarios concerning food insecurity:

“*They [students] need to finish a slushie [frozen sugar-sweetened beverage]] before coming inside the school gates…it’s a bit hard to manage…especially because we’ve got 1600 kids. It’s like hard to monitor what each kid brings in*” (School Personnel #7).

*Sometimes it is a losing battle. Sometimes we’ve had parents drop off fast food. We’ve had to have an uncomfortable conversation that it’s not allowed. But when it’s their only food*,* we’ve had to allow them to eat it in a separate room*” (School Personnel #1).

The delivery of home economics and nutrition education classes was emphasised by several school personnel as a critical strategy to counteract the influence of the external food environment. These subjects were seen as essential for equipping students with the practical skills and knowledge to make healthier food choices, particularly in the context of easy access to unhealthy food option just outside the school gates:

*“I think that the home economics course that we run is quite good at educating on health…teaching students the skills to be able to cook at home. Health [subject] also teaches about the sugar contents [of food] so there is awareness into what they’re putting inside their bodies. I think that the school is doing a reasonable job at educating”* (School Personnel #5).

An additional strategy employed by the school was to provide students with opportunities to participate in after school academies and clubs. This not only assisted in managing the after-school rush to the food outlets, but also served as an opportunity to reinforce some of the health messages learnt in the classroom:

“*We do offer a lot of academies…the seniors have access to the library after school…sports academies and like board games and fitness clubs*…” (School Personnel #2).

## Discussion

This study is, to our knowledge, the first to examine school personnel perspectives on the impact of external food outlets located near schools in Australia. Our findings demonstrate that proximity to external outlets strongly influences students’ access to and purchasing of unhealthy food and drinks. The benefits of having a nearby retail precinct were acknowledged, such as employment opportunities, however, school personnel overwhelmingly described the external school food environment as detrimental to students’ health and behaviour. Importantly, this study found that these negative effects were not limited to students, but extended to the school personnel themselves, school operations and the broader community.

The findings are consistent with a growing body of evidence that shows that the density and proximity of unhealthy food outlets near schools strongly influences young peoples’ dietary habits, which contributes to poor nutrition and increased risk of obesity. Previous quantitative studies have established these associations [2], however this study offers qualitative insights from an Australian perspective. Indeed, school personnel described clear behaviour changes following the construction of the external outlets, including student’s routine congregation before and after school, and the increased consumption of energy drinks and discretionary foods; behaviours rarely observed beforehand.

This study also highlights the influence of marketing, sensory cues and peer dynamics on students’ food purchasing and consumption. School personnel described the pull of food advertising, the smell of food from the outlets and the social aspect of students gathering with peers to purchase and consume food. These findings align with the socio-ecological model of health [21] which highlights that dietary choices are influenced by broader social and environmental contexts, not solely determined by individual knowledge and preferences [22, 23].

A key contribution of this study is its focus on school personnel as informants, whose daily engagement with students positions them uniquely to observe both immediate and cumulative effects of the local food environment [16, 17]. Beyond the impact on students, an important finding was the impact of the outlets on staff themselves. This included the additional workload of enforcing school policies, managing student behaviour and the implications for their own dietary intake; and represents an important area for future research.

At the school level, the negative implications of nearby external food outlets were evident. School personnel reported classroom disruption, disconnect between curriculum messages and the environment, which is consistent with findings from previous studies in Australia, and abroad [3, 24]. The school canteen experienced reduced patronage and adapted by adjusting prices and menu options to compete with nearby outlets. Additionally, staff were required to enforce bans on external food and implement closed-gate policies. Although these initiatives demonstrate resourcefulness, they also highlight the burden of, and the limited influence schools can exert over the external food environment.

The negative effects of external food outlets near schools also extended to the wider community. In this study, the retail precinct became a place for youth congregation, which contributed to loitering, shoplifting and other anti-social behaviour. This strained relationships between the school, local businesses and residents, at times required police involvement and specific governance arrangements.

The policy and practice implications are clear. These finding reinforce the need for stronger urban planning regulations that restrict the development of unhealthy food outlets near schools. International examples, including policies in the UK [25] and South Korea [26], demonstrate that such restrictions can reduce inequalities in weight outcomes [27]. Alongside restrictions, the development of infrastructure, such as libraries and youth centres should be prioritised as alternatives to retail precincts. Cross-sector collaboration between education, health, planning and local government is essential to ensure the external school food environment supports, rather than undermines public health.

This study has some limitations. As it focused on a single school and the sample size was small, findings are not generalisable across all contexts. However, the inclusion of diverse school personnel, from leaders, classroom teachers to canteen staff and youth workers provided valuable depth and breadth of insight. Importantly, this study is the first in Australia to explore school personnel perspectives on the impact of external food outlets located near schools, offering important evidence to guide future research.

## Conclusion

It is clear that the proximity of external food outlets to schools results in challenges with far-reaching consequences for student health, school operations and the broader community. Without structural and policy change, schools will continue to carry a disproportionate burden in mitigating the harms of an unhealthy external food environment.

## Supplementary Information

Supplementary Material 1.

## Data Availability

The datasets used and/or analysed during the current study are available from the corresponding author on reasonable request.

## References

[1] Caraher M, Lloyd S, Mansfield M, Alp C, Brewster Z, Gresham J. Secondary school pupils’ food choices around schools in a London borough: Fast food and walls of crisps. Appetite. 2016;103:208–20. 10.1016/j.appet.2016.04.016. 27105582 10.1016/j.appet.2016.04.016

[2] Caruso OT, McEachern LW, Minaker LM, Gilliland JA. The Influence of the School Neighborhood Food Retail Environment on Unhealthy Food Purchasing Behaviors Among Adolescents: A Systematic Review. J Nutr Educ Behav. 2024;56(3):145–61. 10.1016/j.jneb.2023.11.005. (acccessed 2025/09/17). 38284954 10.1016/j.jneb.2023.11.005

[3] França FCO, Andrade IDS, Zandonadi RP, Sávio KE, Akutsu R. Food Environment around Schools: A Systematic Scope Review. Nutrients. 2022;14(23). DOI: 10.3390/nu14235090 From NLM. 10.3390/nu14235090 PMC9739807 36501120

[4] Smith D, Cummins S, Clark C, Stansfeld S. Does the local food environment around schools affect diet? Longitudinal associations in adolescents attending secondary schools in East London. BMC Public Health. 2013;13(1):70. 10.1186/1471-2458-13-70. 23347757 10.1186/1471-2458-13-70 PMC3567930

[5] Smets V, Vandevijvere S. Changes in retail food environments around schools over 12 years and associations with overweight and obesity among children and adolescents in Flanders, Belgium. BMC Public Health. 2022;22(1):1570. 10.1186/s12889-022-13970-8. 35982440 10.1186/s12889-022-13970-8 PMC9387020

[6] Olarte DA, Petimar J, James P, Cooksey-Stowers K, Cash SB, Rimm EB, et al. Trends in Quick-Service Restaurants near Public Schools in the United States: Differences by Community, School, and Student Characteristics. J Acad Nutr Dietetics. 2023;123(6):923–e321. 10.1016/j.jand.2023.01.016. 10.1016/j.jand.2023.01.016 PMC10200734 36740187

[7] Tse K, Zeng MX, Gibson AA, Partridge SR, Raeside R, Valanju R, et al. Retrospective analysis of regional and metropolitan school food environments using Google Street View: A case study in New South Wales, Australia with youth consultation. Health Promot J Austr. 2025;36(2):e930. 10.1002/hpja.930. From NLM. 39415435 10.1002/hpja.930 PMC11806403

[8] Trapp GSA, Hooper P, Billingham W, Thornton L, Sartori A, Kennington K, et al. Would you like fries with that? Investigating fast-food outlet availability near schools in Perth, Western Australia. Health Promotion J Australia. 2023;34(1):85–90. 10.1002/hpja.682. From NLM. 10.1002/hpja.682 PMC10108019 36433680

[9] Thornton LE, Lamb KE, Ball K. Fast food restaurant locations according to socioeconomic disadvantage, urban–regional locality, and schools within Victoria, Australia. SSM - Popul Health. 2016;2:1–9. 10.1016/j.ssmph.2015.12.001. 29349122 10.1016/j.ssmph.2015.12.001 PMC5757894

[10] Coffee NT, Kennedy HP, Niyonsenga T. Fast-food exposure around schools in urban Adelaide. Public Health Nutr. 2016;19(17):3095 – 105. DOI: 10.1017/s1368980016001385 From NLM. 10.1017/S1368980016001385 PMC10270798 27297639

[11] Trapp GSA, Hooper P, Thornton L, Kennington K, Sartori A, Hurworth M, et al. Association between food-outlet availability near secondary schools and junk-food purchasing among Australian adolescents. Nutrition. 2021;91–92:111488. 10.1016/j.nut.2021.111488. From NLM. 10.1016/j.nut.2021.111488 34626957

[12] Patton GC, Sawyer SM, Santelli JS, Ross DA, Afifi R, Allen NB, et al. Our future: a Lancet commission on adolescent health and wellbeing. Lancet. 2016;387(10036):2423–78. 10.1016/S0140-6736(16)00579-1. (acccessed 2025/01/21). 27174304 10.1016/S0140-6736(16)00579-1 PMC5832967

[13] Green EM, Spivak C, Dollahite JS. Early adolescent food routines: A photo-elicitation study. Appetite. 2021;158:105012. 10.1016/j.appet.2020.105012. 33132192 10.1016/j.appet.2020.105012

[14] da Costa Peres CM, Gardone DS, Costa BVL, Duarte CK, Pessoa MC, Mendes LL. Retail food environment around schools and overweight: a systematic review. Nutr Rev. 2020;78(10):841–56. 10.1093/nutrit/nuz110. From NLM. 31968100 10.1093/nutrit/nuz110

[15] Pineda E, Bascunan J, Sassi F. Improving the school food environment for the prevention of childhood obesity: What works and what doesn’t. Obes Rev. 2021;22(2):e13176. 10.1111/obr.13176. From NLM. 33462933 10.1111/obr.13176

[16] Nollen NL, Befort CA, Snow P, Daley CM, Ellerbeck EF, Ahluwalia JS. The school food environment and adolescent obesity: qualitative insights from high school principals and food service personnel. Int J Behav Nutr Phys Activity. 2007;4(1):18. 10.1186/1479-5868-4-18. 10.1186/1479-5868-4-18 PMC1892033 17511873

[17] González-Salgado IdL, Rivera-Navarro J, Díez J, Gravina L. School principals’ perceptions of adolescents’ eating behaviors in two Spanish cities: a qualitative study based on the neo-ecological theory. Appetite 2025;212:108013. 10.1016/j.appet.2025.108013. 10.1016/j.appet.2025.108013 40252810

[18] Devine LD, Gallagher AM, Briggs S, Hill AJ. Factors that influence food choices in secondary school canteens: a qualitative study of pupil and staff perspectives. Frontiers in Public Health 2023;11, Original Research. 10.3389/fpubh.2023.1227075. 10.3389/fpubh.2023.1227075 PMC10375012 37522007

[19] Guest G, MacQueen K, Namey E. Applied Thematic Analysis. Thousand Oaks, California: SAGE Publications, Inc.; 2012. Available from: https://methods.sagepub.com/book/mono/applied-thematic-analysis/toc.

[20] Tong A, Sainsbury P, Craig J. Consolidated criteria for reporting qualitative research (COREQ): a 32-item checklist for interviews and focus groups. Int J Qual Health Care. 2007;19(6):349–57. 10.1093/intqhc/mzm042. (acccessed 11/7/2025). 17872937 10.1093/intqhc/mzm042

[21] Story M, Kaphingst KM, Robinson-O’Brien R, Glanz K. Creating healthy food and eating environments: policy and environmental approaches. Annu Rev Public Health. 2008;29:253–72. 10.1146/annurev.publhealth.29.020907.090926. From NLM. 18031223 10.1146/annurev.publhealth.29.020907.090926

[22] Neufeld LM, Andrade EB, Ballonoff Suleiman A, Barker M, Beal T, Blum LS, et al. Food choice in transition: adolescent autonomy, agency, and the food environment. Lancet. 2022;399(10320):185–97. 10.1016/S0140-6736(21)01687-1. (acccessed 2025/01/21). 34856191 10.1016/S0140-6736(21)01687-1

[23] Ziegler AM, Kasprzak CM, Mansouri TH, Gregory AM, Barich RA, Hatzinger LA, Leone LA, Temple JL. An Ecological Perspective of Food Choice and Eating Autonomy Among Adolescents. Frontiers in Psychology 2021;12, Original Research. 10.3389/fpsyg.2021.654139. 10.3389/fpsyg.2021.654139 PMC8097152 33967917

[24] Gingell T, Esdaile E, Gallegos D. School food and nutrition environments in Australian primary schools: A scoping review. PLoS ONE. 2025;20(7). 10.1371/journal.pone.0327310. 10.1371/journal.pone.0327310 PMC12212514 40591617

[25] Brown H, Xiang. Huasheng, Albani, Viviana, Goffe, Louis, Akhter, Nasima, Lake, Amelia, Sorrell, Stewart, Gibson, Emma, Wildman, John,. No new fast-food outlets allowed! Evaluating the effect of planning policy on the local food environment in the North East of England. Soc Sci Med. 2022;306:115126. 10.1016/j.socscimed.2022.115126. 35724588 10.1016/j.socscimed.2022.115126

[26] Ministry of Food and Drug Safety. Promoting Healthy Diets and Safe Food Consumption;, 2019. https://www.mfds.go.kr/eng/wpge/m_13/de011004l001.do (accessed on 28 March 2024).

[27] Xiang H, Goffe AA, Brown H. Planning policies to restrict fast food and inequalities in child weight in England: a quasi-experimental analysis. Obesity 2024;32(12):2345-53. 10.1002/oby.24127. 10.1002/oby.24127 PMC11589531 39439285

