# Supplementary material for: Temptation at the school fence: a qualitative exploration of the impact of external food outlets on the school community
Source: BMC Public Health. 2026 Mar 13;26:1304. doi: 10.1186/s12889-026-26917-0 (PMC13101357; doi:10.1186/s12889-026-26917-0)
Supplement: Supplementary file 1 — Supplementary Material 1. [file 12889_2026_26917_MOESM1_ESM.pdf]

The Food Environment Around Schools sTudy (FEAST)

School Personnel Interview Guide

Time & Date: ___________________________FEAST Interviewer: ________________________________

Introduction [5 minutes]:

- Welcome and thank you for participating in our interview today.
- My name is <state name> and I work at the Telethon Kids Institute. Our research team is investigating food outlet availability near schools and the influence it has on the dietary intake, food preferences and food purchasing behaviours of students, including use of the school canteen.
- The purpose of this interview today is to discuss your thoughts and experiences relating to the food environment within your school as well as outside of your school.
- Anything you say today is strictly confidential – your name will not be used in any reports arising from this interview or other aspects of the research.
- Your participation in our discussion today is voluntary. You don't have to answer any questions that make you uncomfortable and we can stop the interview at any time.
- I'd like to record our conversation so I can accurately recall what we've spoken about. The recording will be stored securely at the Telethon Kids Institute for at least seven years before being destroyed. Do I have your permission to record our conversation today?
- Our discussion today should take around 30 minutes. Please stop me if you have any questions to ask or points to make.
- Is there anything you'd like to ask me now? Are you happy for me to continue?

Interview questions

1. Before we begin, we need to gather some background information about each study participant so we can describe the study sample and ensure we're capturing different perspectives. You don't have to answer these questions if you prefer not to.
   Can you tell me your gender (male, female, non-binary, prefer not to say, other)?
2. What is your current role at the school?
3. How long have you been affiliated with this school?
4. Can you tell me about the food related behaviour of students at your school? Things like what you notice they eat and drink and whether that impacts their behaviour?
5. I've noticed there are lots of takeaway food places around your school, how do you feel about that? (prompt for positive aspects and negative aspects, their use, thoughts about the different types of food outlets)
6. I've noticed there are lots of outdoor advertisements for takeaway food and drink around your school, how do you feel about that?
7. Do you have any thoughts about how school children can be supported to find healthy food options or make healthy food choices? (prompt for what schools, local council, government could do)

Closing instructions:

8. Do you have anything else to say about the topics raised today?
   - Thank them for their time.
